# Comparing Gut Microbial Composition and Functional Adaptations between SPF and Non-SPF Pigs

**DOI:** 10.4014/jmb.2402.02018

**Published:** 2024-05-30

**Authors:** Haesun Lee, Woncheoul Park, Jingu No, Nam Woong Hyung, Ju-Yeong Lee, Seokho Kim, Hyeon Yang, Poongyeon Lee, Eunju Kim, Keon Bong Oh, Jae Gyu Yoo, Seunghoon Lee

**Affiliations:** 1 Animal Biotechnology Division, National Institute of Animal Science, Rural Development Administration, Wanjugun, Jeollabuk-do 55365, Republic of Korea; 2 Animal Genomics and Bioinformatics Division, National Institute of Animal Science, Rural Development Administration, Wanju-gun, Jeollabuk-do 55365, Republic of Korea; 3 Hanwoo Research Institute, National Institute of Animal Science, Rural Development Administration, Wanju-gun, Jeollabuk-do 55365, Republic of Korea

**Keywords:** Gut microbiome, specific pathogen free, miniature pig

## Abstract

The gut microbiota is a key factor significantly impacting host health by influencing metabolism and immune function. Its composition can be altered by genetic factors, as well as environmental factors such as the host's surroundings, diet, and antibiotic usage. This study aims to examine how the characteristics of the gut microbiota in pigs, used as source animals for xenotransplantation, vary depending on their rearing environment. We compared the diversity and composition of gut microbiota in fecal samples from pigs raised in specific pathogen-free (SPF) and conventional (non-SPF) facilities. The 16S RNA metagenome sequencing results revealed that pigs raised in non-SPF facilities exhibited greater gut microbiota diversity compared to those in SPF facilities. Genera such as *Streptococcus* and *Ruminococcus* were more abundant in SPF pigs compared to non-SPF pigs, while Blautia, Bacteroides, and Roseburia were only observed in SPF pigs. Conversely, *Prevotella* was exclusively present in non-SPF pigs. It was predicted that SPF pigs would show higher levels of processes related to carbohydrate and nucleotide metabolism, and environmental information processing. On the other hand, energy and lipid metabolism, as well as processes associated with genetic information, cell communication, and diseases, were predicted to be more active in the gut microbiota of non-SPF pigs. This study provides insights into how the presence or absence of microorganisms, including pathogens, in pig-rearing facilities affects the composition and function of the pigs' gut microbiota. Furthermore, this serves as a reference for tracing whether xenotransplantation source pigs were maintained in a pathogen-controlled environment.

## Introduction

Pigs are frequently used in biomedical research because they are similar to humans in terms of genetics, physiology, and anatomy, with the advantages of short generation intervals, large litter sizes, and early sexual maturity [1]. Compared to small laboratory animals like rodents, the pathophysiological and immunological responses of pigs more closely resemble those of humans [2]. With these characteristics, pigs are considered optimal animal models of infectious, metabolic, neurodegenerative, and genetic diseases of humans and even sources of biological materials for xenotransplantation [1, 3, 4]. For pigs to be utilized in xenotransplantation, comprehensive documentation of all aspects of pig characteristics, including origin, genetic characteristics, rearing environment, and the history of potential exposure to pathogens, is imperative [5, 6].

Among these various characteristics of pigs produced and managed for xenotransplantation, we have focused on the analysis of the gut microbiota. The gut microbiota is recognized as a significant factor often referred to as the “second genome”, closely linked with digestion, metabolism, immunity, and diseases in the host [7]. It also has a great influence on productivity in livestock [8]. The abrupt change in gut microbiota is considered a causative factor for functional and inflammatory gastrointestinal (GI) disorders in all animals, including piglets, during the weaning transition [9, 10]. Thus, considering that the interaction between the microbiota and the host’s immune mechanisms influences tissue homeostasis and protection from pathogens, the gut microbiota of the host can serve as an important indicator for understanding the host’s physiological characteristics [11].

The composition and diversity of the gut microbiota are influenced by not only the genetic variation of the host but also by environmental factors [12]. Numerous studies have reported interactions between alterations in the gut microbiota and various environmental factors, such as dietary changes, diseases, and pathogenic microorganisms [13-16]. The rearing environment is also a noteworthy factor that can induce changes in the composition of gut microbiota. In chickens, differences in gut microbiota composition were observed between cage rearing and free-range rearing [10]. Yu *et al*. investigated differences in intestinal microflora in stool samples from both specific pathogen-free (SPF) and non-SPF beagle dogs according to their age [7]. The analysis of gut microbiota composition could serve as a tool for health monitoring by identifying undesirable pathogens in animals [17]. The influence of environments harboring a variety of microorganisms, including pathogens, on the gut microbiota of animals could be elucidated through comparisons between pathogen-free and conventional facilities.

Here, we aimed to compare the differences in the composition of gut microbiota in pigs based on the presence or absence of pathogens in the breeding environment. By predicting the functions of the gut microbiota, we sought to determine how these functions, particularly those related to defense mechanisms against pathogens, vary depending on the rearing environment. This study primarily aims to understand the characteristics of the gut microbiota in pigs based on their rearing environment. Secondly, by utilizing the differences in the composition of gut microbiota according to the environment, we aim to establish criteria for predicting whether xenotransplantation pigs have been exposed to pathogens.

## Materials and Methods

### Animals and Experimental Design

All experiments involving animals were approved by the Institutional Animal Care and Use Committee of the National Institute of Animal Science (Approval No: NIAS2020-0473). A total of 8 Massachusetts General Hospital (MGH) miniature pigs (4 SPF and 4 non-SPF pigs) from National Institute of Animal Science were enrolled into this study. These pigs suppressed the expression of the α1,3-galactosyltransferase (GGTA) gene, involved in the synthesis of the galactose-α1,3-galactose (Gal) antigen, and overexpressed the membrane cofactor protein (MCP, CD46), a complement regulatory protein, to control xenograft rejection [18].

The SPF facility employs high efficiency particulate air (HEPA) filters to introduce filtered air, preventing exposure to animals in the general environment and thereby mitigating the risk of pathogens that could infect pigs. In order to support SPF pig production and management, the facility conducts regular testing for 42 types of pathogens. SPF pigs were bred in sterilized isolator (the positive pressure of 5 mmH_2_O, humidity of 50%) and fed artificially sterilized milk. The temperature was maintained at 38°C for the first week and then decreased by 3°C every week until 5th week after birth. After weaning, they were raised in the SPF level breeding cage with sterilized pellet-type commercial feed and sterilized water. Non-SPF pigs were naturally fed by the sow until 5 weeks of age and then gradually weaned in conventional facility. The fecal samples were obtained from SPF and non-SPF pigs at the age of 18 months. These samples were collected immediately after spontaneous defecation and frozen at -80°C.

### DNA Extraction and Sequencing

DNA was extracted from the stool samples and 16S amplicon libraries were constructed using Herculase II Fusion DNA Polymerase Nextera XT Index Kit V2 (Illumina, USA) through the services provided by GnC Bio Co.(Republic of Korea). Sequences of 16S rRNA at V3 and V4 region were targeted to amplify. The quality and quantity of amplicon libraries were assessed using D1000 ScreenTape (Agilent, USA) and Picogreen (Agilent), respectively. Metagenome sequencing was performed using an illumina Miseq sequencer platform (Illumina). To ensure data quality, the low-quality reads (Q < 30) were removed from the raw sequencing reads. Raw sequence data were submitted to NCBI Gene Expression Omnibus (GEO) and deposited with GEO accession number GSE264183.

### Taxonomy Classification, Microbial Functional Prediction and Statistical Analysis

The classification of taxonomic abundance was conducted using the Quantitative insights into microbial ecology 2 (QIIME2, 2022.8.0.) [19]. DADA2 was used for quality filtering and denoising, low-quality sequences were removed with a quality score (< Q 25). The assigned taxonomy IDs were aligned based on the Greengenes (v.13_8) reference database. To evaluate alpha diversity, number of observed species, pielou’s, and Shannon indexes were assessed using QIIME2 followed by Kruskal-Wallis test to detect statistical differences (*p*-value < 0.05). Beta diversity was measured by unweighted UniFrac that is standard multivariate statistical techniques including principal coordinate analysis (PCoA). The significant differences of gut microbial communities between SPF and non-SPF pigs were assessed by permutational multivariate analysis of variance (PERMANOVA). The relative abundance of specific bacterial taxa in gut microbiota between two groups was identified using linear discriminant analysis (LDA) effect size (LEfSe) method [20]. An LDA score of more than 4 was considered statistically significant taxa. Microbial function was predicted using Phylogenetic Investigation of Communities by Reconstruction of Unobserved States2 (PICRUSt2, v2.5.1) [21] plugin in QIIME2. The predicted functions were aligned to Kyoto encyclopedia of genes and genomes (KEGG) database. KEGG Orthology (KO) and the effect size of KOs were measured using QIIME2 picrust2 full-pipeline. Using the ALDEx2 [22] package in R (v1.32), a comparative analysis between two groups was conducted. The distinct KOs that were identified using effect size threshold > 1 and/or Benjamin-Hochberg adjusted *p*-value < 0.01 (Wilcoxon rank sum test). Gene set enrichment analysis (GSEA) [23] with the cluster-profiler from R package was applied to determine the significant enriched metabolic pathway in the KO database for distinct KOs functions. The LEfSe and KEGG were visualized using ggplot2 in R package (R Foundation for statistical Computing, Austria).

## Results

### The Gut Microbiota of Non-SPF Pigs Was More Diverse Than That of SPF Pigs

To identify potential differences in the gut microbiota composition between SPF and non-SPF pigs, 16S rRNA metagenome sequencing was performed using microbial DNA extracted from a total of 8 stool samples of pigs in the SPF facility (*n* = 4) and the non-SPF facility (*n* = 4) at the age of 18 months. Initially, a total of 1,889,964 raw sequencing reads were generated. Following the elimination of low-quality reads with a quality score of less than 30, a total of 1,818,452 reads were obtained for analysis. These reads ranged from 216,030 to 247,648 reads, with an average of approximately 227,307 reads per sample. The information on raw data (Table S1) and pre-processed data (Table S2) has been provided.

**Fig. 1:**
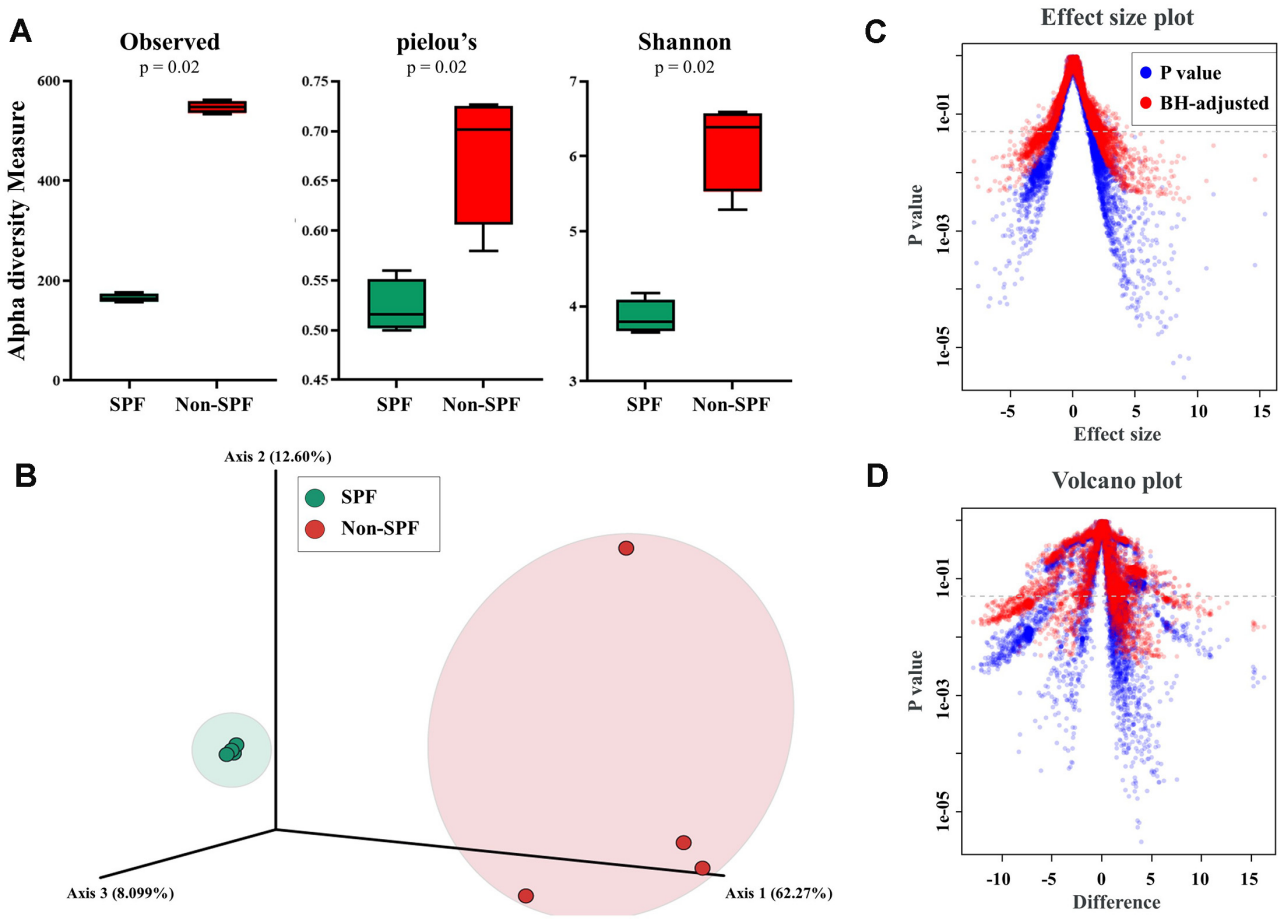
Comparisons of alpha and beta diversity of gut microbiota between SPF and non-SPF pigs. (**A**) The alpha diversity was compared by assessing the number of observed species and the Pielou’s and Shannon indices. (**B**) Biogeography of gut microbiota represented on principal coordinates analysis (PCoA) plots in a three-dimensional view. Each symbol on the plot indicates an individual gut microbiome. (**C**) Effect size plot. (**D**) Volcano plot.

The alpha diversity of SPF pigs was significantly lower than that of non-SPF pigs, as determined through the number of observed species, Pielou’s index, and Shannon index (Fig. 1A; Kruskal‒Wallis test). Next, we examined the dissimilarity in community structure between the SPF and non-SPF groups. The PCoA plot showed significant differences in gut microbial communities between non-SPF and SPF pigs in a three-dimensional view, with the gut microbiota of SPF pigs exhibiting dispersion, while that of non-SPF pigs formed distinct clusters (Fig. 1B). An effect size plot illustrates the magnitude of the effect size for each microbial feature potted against its corresponding P value (Fig. 1C). A volcano plot simultaneously displays the differences in microbial abundance and their statistical significance (Fig. 1D). In this study, data significance was determined after adjustment using Benjamini-Hochberg (BH) procedure.

### The Gut Microbial Composition Differentially Represented in SPF and non-SPF Pigs

**Fig. 2:**
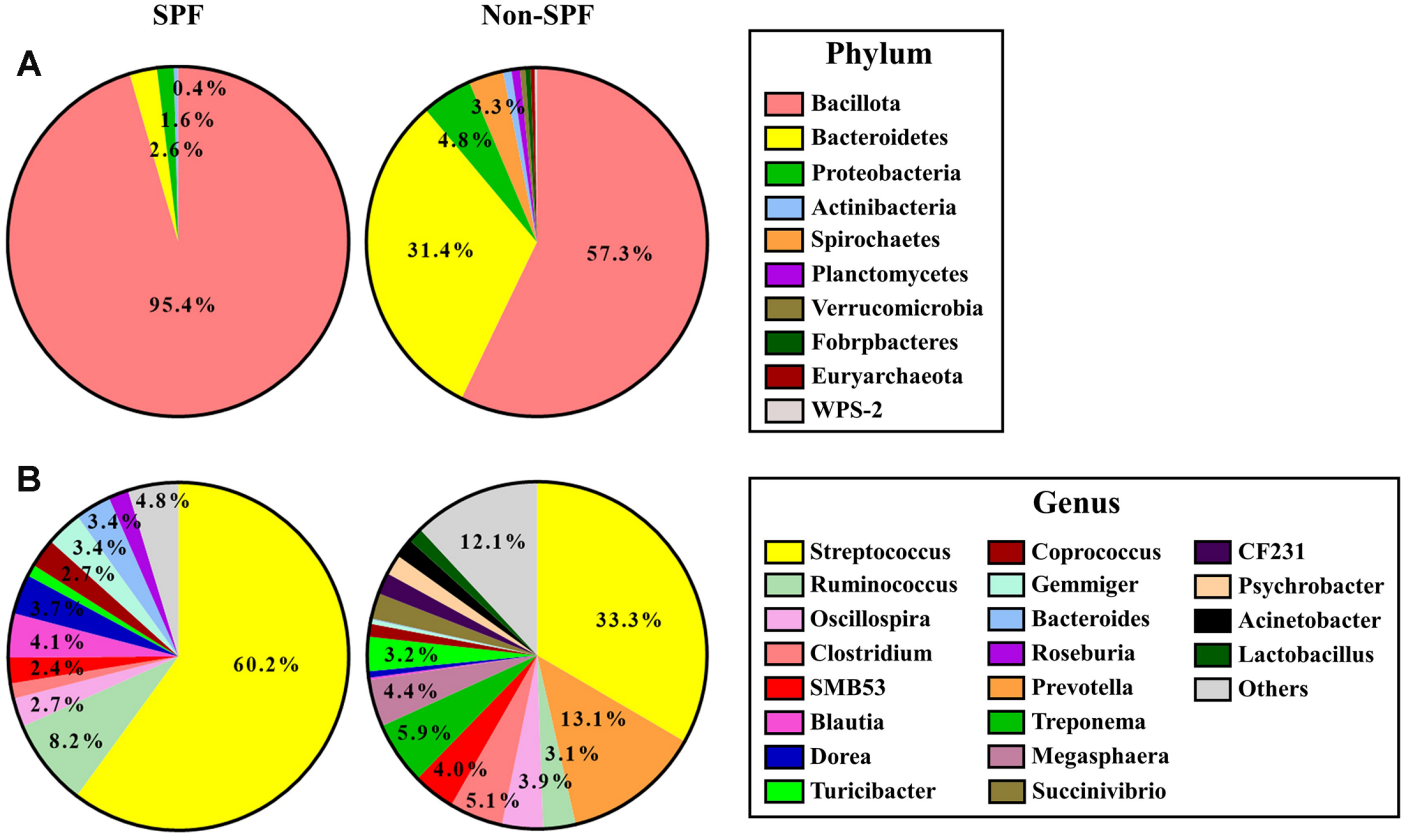
Community composition of gut microbiota in SPF and non-SPF pigs. The graph represents the average percentage of community abundance at the (**A**) phylum and (**B**) genus levels in SPF (left) and non-SPF (right) pigs.

We compared the composition of the gut microbiota at the phylum or genus level between the two groups. At the phylum level, Bacillota were the most dominant, representing 95.4% and 57.3% of all bacterial populations in SPF and non-SPF, respectively. Although the non-SPF group exhibited notably higher values compared to the SPF group, *Bacteroidetes* constituted the second most abundant phylum both in SPF (2.6%) and non-SPF (31.4%). In the SPF group, *Proteobacteria* (1.6%) and *Actinobacteria* (0.4%) followed, while in the non-SPF group, *Proteobacteria* (4.8%), *Spirochaetes* (3.3%), *Planctomycetes* (0.8%), and *Euryarchaeota* (0.4%) occupied subsequent positions (Fig. 2A).

At the genus level, *Streptococcus* was most dominant in both SPF (60.2%) and non-SPF (33.3%) pigs. In SPF pigs, following *Streptococcus*, the genera *Ruminococcus* (8.3%), *Blautia* (4.1%), Dorea (3.7%), Gemmiger (3.4%), and Bacteroides (3.4%) were observed as the subsequent dominant taxa. In non-SPF pigs, *Prevotella* (13.1%), Treponema (5.9%), Clostridium (5.1%), Megasphaera (4.4%), SMB53 (4.0%), Oscillospira (3.9%), Turicibacter (3.2), and *Ruminococcus* (3.1%) were identified as the subsequent dominant genera, following *Streptococcus*. The genera *Blautia*, *Bacteroides*, and *Roseburia* (1.9%) were present exclusively in the gut microbiota of non-SPF pigs, while *Prevotella*, *Treponema*, *succinivibrio* (2.4%), *CF231* (2%), *Phychrobacter* (1.9%), and *Acinetobacter* (1.8%) were found uniquely in the gut microbiota of SPF pigs (Fig. 2B).

**Fig. 3:**
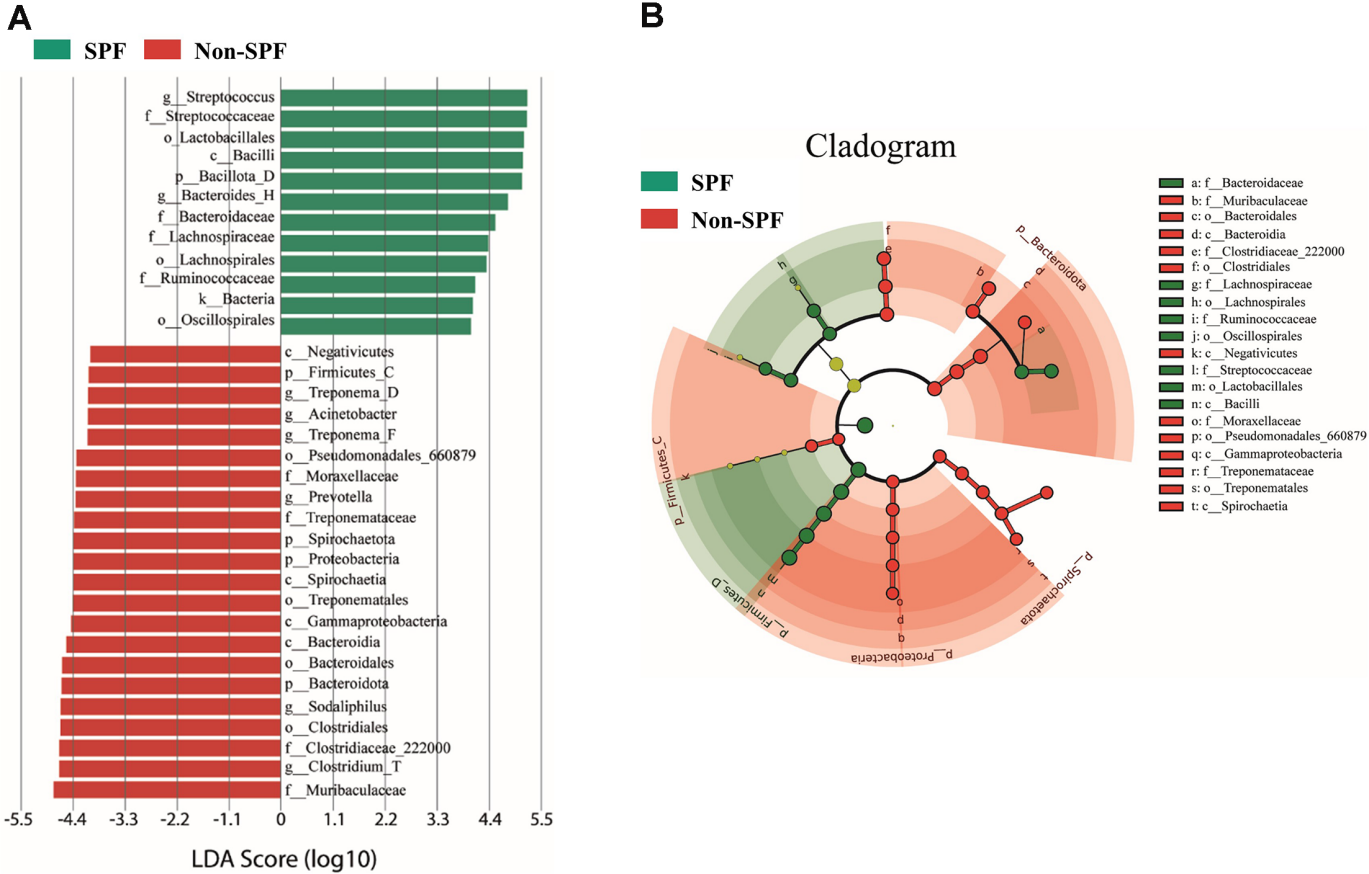
Relative abundance of bacterial taxa in the gut microbiota between SPF and non-SPF pigs analyzed using linear discriminant analysis (LDA) effect size (LEfSe). (**A**) Statistically significant differences are indicated by an LDA score of more than 4.0. (**B**) The cladogram shows the phylogenetic distribution of gut microbiota.

To investigate variations in specific bacterial taxa between SPF and non-SPF pigs, we performed linear discriminant analysis (LDA) effect size (LEfSe) analysis. Significant differences in the relative distribution of 34 bacterial taxa were observed between SPF and non-SPF pigs (LDA score > 4.0, *P* < 0.05). Among them, 12 bacterial taxa, 12 bacterial taxa were more prevalent in SPF pigs; this group including four families–*Streptococcaceae*, *Bacteroidaceae*, *Lachnospiraceae*, *Ruminococcaceae*–and two genera, *Streptococcus* and *Bacteroides_H*. Meanwhile, 22 bacterial taxa, which include four families–*Muribaculaceae*, *Clostridiaceae_222000*, *Treponemataceae*, *Moraxellaceae*–and six genera–*Clostridium_T*, *Sodaliphilus*, *Prevotella*, *Treponema_F*, *Actinetobacter*, and *Triponema_D*–were significantly abundant in the non-SPF group (Fig. 3). Result of LEfSe analysis with an LDA score of 3 or higher are presented in Fig. S1.

### The Functional Potential in Metabolic and Biological Processes Exhibited Differences between SPF and Non-SPF Pigs

**Fig. 4:**
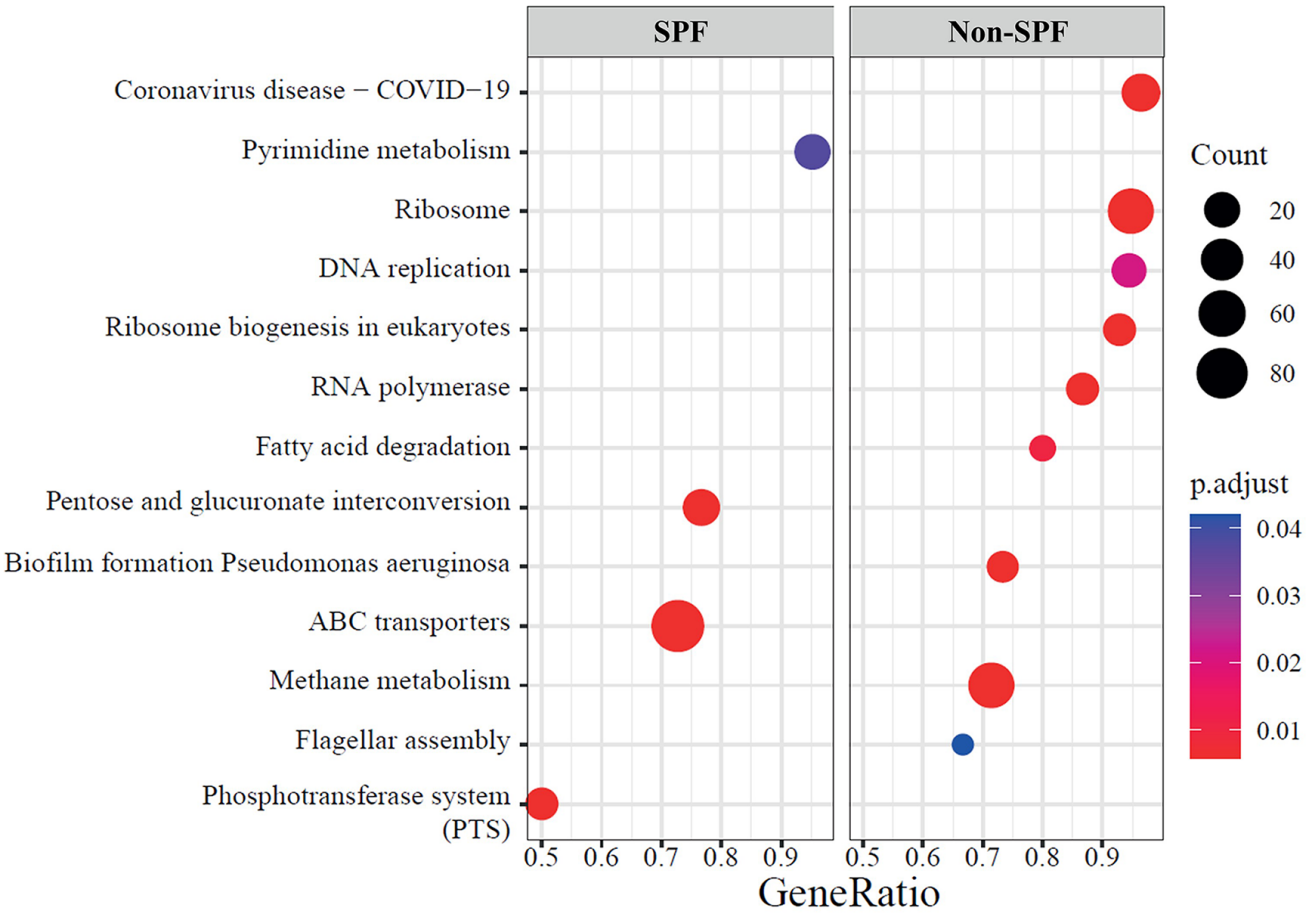
Enriched gut microbial function in SPF and non-SPF pigs. Dot plot diagram showing the enriched KEGG pathways.

To further predict functional differences in metabolic and biological processes between SPF and non-SPF pigs, we performed microbial functional profiling. The bacterial genes involved in the metabolism of carbohydrates (pentose and glucoronate interconversion) and nucleotides (pyrimidine metabolism) were predicted to be enriched in SPF pigs, while those of energy (methane metabolism) and lipids (fatty acid degradation) were predicted to be enriched in non-SPF pigs. In SPF pigs, the main presented processes were membrane transport (ATP binding cassette transporters, phosphotransferase system) as environmental information processes. The enriched processes observed in non-SPF pigs were related to genetic information processes, including transcription (RNA polymerase), translation (ribosome biogenesis in eukaryotes, ribosome), replication and repair (DNA replication), cellular processes such as cellular community (biofilm formation–*Pseudomonas aeruginosa*) and cell motility (flagellar assembly), and diseases (coronavirus disease–COVID-19) (Fig. 4).

## Discussion

Growing interest in gut microbiota has prompted research into their interactions with hosts in various contexts, including environmental changes and diseases [12-16]. Recent studies have particularly focused on the role of gut microbiota in organ transplantation and their influence on immune regulation in transplant recipients [24, 25]. This focus is due to the critical role that gut microbiota play in modulating the host’s immune system, metabolism, and various physiological functions. Analyzing the characteristics of gut microbiota in pigs, used as source animals in xenotransplantation, provides essential data for understanding these source animals. Specifically, insights into the gut microbiota under pathogen-controlled conditions are crucial for xenotransplantation research. However, the distinctions in gut microbiota between miniature pigs reared in pathogen-free versus conventional facilities remain unclear. In this study, we characterized the compositions and functions of the gut microbiota in SPF and non-SPF miniature pigs.

We investigated the differences in the gut microbiota composition between SPF and non-SPF pigs. The composition of the gut microbiota was more abundant in non-SPF pigs than in SPF pigs. We further observed that the gut microbiota of non-SPF and SPF pigs formed distinct clusters, indicating a clear separation in microbiota community structures between the two groups. The effect size pot and volcano plot elucidate distinct microbial profile disparities between the groups under study (Fig. 1). Previous research using various breeds of domestic pigs and wild pigs also showed that microbial diversity is lower in SPF pigs than in pigs exposed to a risk of diseases such as African swine fever; this suggests that the diversity of the gut microbiota is influenced by the rearing environment, particularly by microorganisms present in the facilities, feed, water, and other cohabiting individuals [26]. It can be inferred that pigs raised in environments containing a variety of microorganisms, including pathogenic bacteria, would likely exhibit diverse gut microbiota compositions.

The results from the analysis of gut microbiota taxonomic profiles and LEfSe indicated substantial disparities in the gut microbiota composition between SPF and non-SPF pigs. *Prevotella*, identified as one of the most predominant genera in the pig intestine, plays a pivotal role in the synthesis of short-chain fatty acids (SCFAs), a source of energy, along with *Alloprevotella* [7, 27-29]. Nevertheless, in our study, *Prevotella* was present exclusively in the non-SPF pigs, not in SPF pigs. *Alloprevotella* was indeed more abundant in the non-SPF pigs than in the SPF pigs (Figs. 2B and 3). These results are consistent with the previous observation that *Prevotella* and *Alloprevotella* were present exclusively in non-SPF beagles, not in SPF beagles. Yu *et al*. suggested that the presence of *Prevotella* and *Alloprevotella* in non-SPF beagles may offer protection against complex microbial environments with potential anti-inflammatory capabilities [7].

Meanwhile, in SPF pigs, the analysis indicates a higher proportion of alternative microorganisms substituting for the functions of *Prevotella* and *Alloprevotella*. Gorvitovskaia *et al*. elucidated an inverse relationship between the ratio of *Prevotella* and *Bacteroides* in the human gut microbiota [30]. The bacterial taxa mediating SCFA production, such as the family *Lachnospiraceae* [31], genera *Streptococcus* [29], and *Ruminococcus* [32] were more abundant in SPF pigs (20.0%, 60.2%, and 8.3%, respectively) than in non-SPF pigs (4.9%, 33.3%, and 3.1%, respectively). Moreover, the genera *Blautia* [31, 33], *Bacteroides* [33, 34], and *Roseburia* [16, 35] were exclusively found in SPF pigs (4.1%, 3.4%, and 1.9%, respectively). However, the association between a low proportion of *Prevotella* in the gut microbiota and the pathogen-free environment requires further confirmation. The functional predictions of gut microbiota revealed that environmental information processes, such as ABC transporters and the phosphotransferase system, were more abundant in SPF pigs (Fig. 4). The functional processes of membrane transport, which are involved in the absorption of essential nutrients and the expulsion of toxic substances, are modulated in response to environmental changes [36]. These results suggest that pigs raised in a pathogen-free environment, in particular, may exhibit heightened sensitivity to even minor environmental changes. On the other hand, as expected, the cellular processes counteracting pathogenic microorganisms such as *Pseudomonas aeruginosa* and *coronavirus*, were predicted to be more abundant in non-SPF pigs. Additionally, the genetic information processes were predicted to be enriched in non-SPF pigs compared to SPF pigs (Fig. 4). These results are likely attributed to interactions with various environmental microorganisms, including pathogens.

Through this study, we compared the gut microbiota of 18-month-old pigs in a pathogen controlled environment and a conventional environment. As anticipated, the gut microbiota in pigs from the conventional environment exhibited greater diversity, and the functional response to external elements, including pathogens, was more active in conventional pigs compared to those in the pathogen-controlled environment. These findings underline notable disparities in gut microbial diversity and composition between SPF and non-SPF pig facilities. Understanding these differences could offer insights into pig health and pathology. In xenotransplantation, pathogens are a crucial factor influencing the survival of immunocompromised recipients of xenografts. If alterations in gut microbiota serve as indicators of the influx history of pathogens from xenotransplantation donor pigs, it is anticipated that stable outcomes can be expected during xenotransplantation. Therefore, it is speculated that stable outcomes can be expected even in xenotransplantation when pathogens are controlled. Moreover, this knowledge might aid in enhancing pig farming environments and managing diseases more effectively in the future. Subsequent investigations may be needed to examine the changes in gut microbiota composition based on the age of the pigs.

## Supplemental Materials

Supplementary data for this paper are available on-line only at http://jmb.or.kr.
