# Supplementary material for: Comparing Gut Microbial Composition and Functional Adaptations between SPF and Non-SPF Pigs
Source: J Microbiol Biotechnol. 2024 May 30;34(7):1484–90. doi: 10.4014/jmb.2402.02018 (PMC11294643; doi:10.4014/jmb.2402.02018)
Supplement: Supplementary file 1 [file jmb-34-7-1484-supple.pdf]

J. Microbiol. Biotechnol. 2024. 34(7): 1484–1490
https://doi.org/10.4014/jmb.2402.02018

Supplementary Tables and Figure

Comparing Gut Microbial Composition and Functional Adaptations between SPF and Non-SPF pigs

Supplementary Table S1. Raw data.

Sample	Total read bases (bp)	Total reads	GC (%)	AT (%)	Q20 (%)	Q30 (%)
SPF-A	71,386,966	237,166	52.5	47.5	91.2	82.1
SPF-B	77,866,292	258,692	52.9	47.1	91.5	82.8
SPF-C	73,429,552	243,952	52.4	47.6	91.3	82.4
SPF-D	74,948,398	248,998	52.4	47.6	91.9	83.2
Non-SPF-E	67,353,566	223,766	53.6	46.4	91.3	82.2
Non-SPF-F	70,401,492	233,892	53.8	46.2	91.2	82.2
Non-SPF-G	69,958,420	232,420	54.2	45.8	91.0	81.9
Non-SPF-H	63,534,478	211,078	53.4	46.6	91.3	82.4
Sum	568,879,164	1,889,964	-	-	-	-
Average	71,109,896	236,246	53.2	46.9	91.3	82.4

Supplementary Table S2. Pre-processed data.

Sample	Pre-processed read bases (bp)	Pre-processed reads	Gbp
SPF-A	57,961,399	227,172	0.06
SPF-B	63,831,115	247,648	0.06
SPF-C	60,070,146	234,554	0.06
SPF-D	62,130,336	240,580	0.06
Non-SPF-E	55,046,902	216,030	0.06
Non-SPF-F	57,521,476	224,942	0.06
Non-SPF-G	56,740,997	223,872	0.06
Non-SPF-H	51,930,307	203,654	0.05
Sum	1,818,452	465,232,678	0.47
Average	227,307	58,154,085	0.06

Supplementary Figure S1.

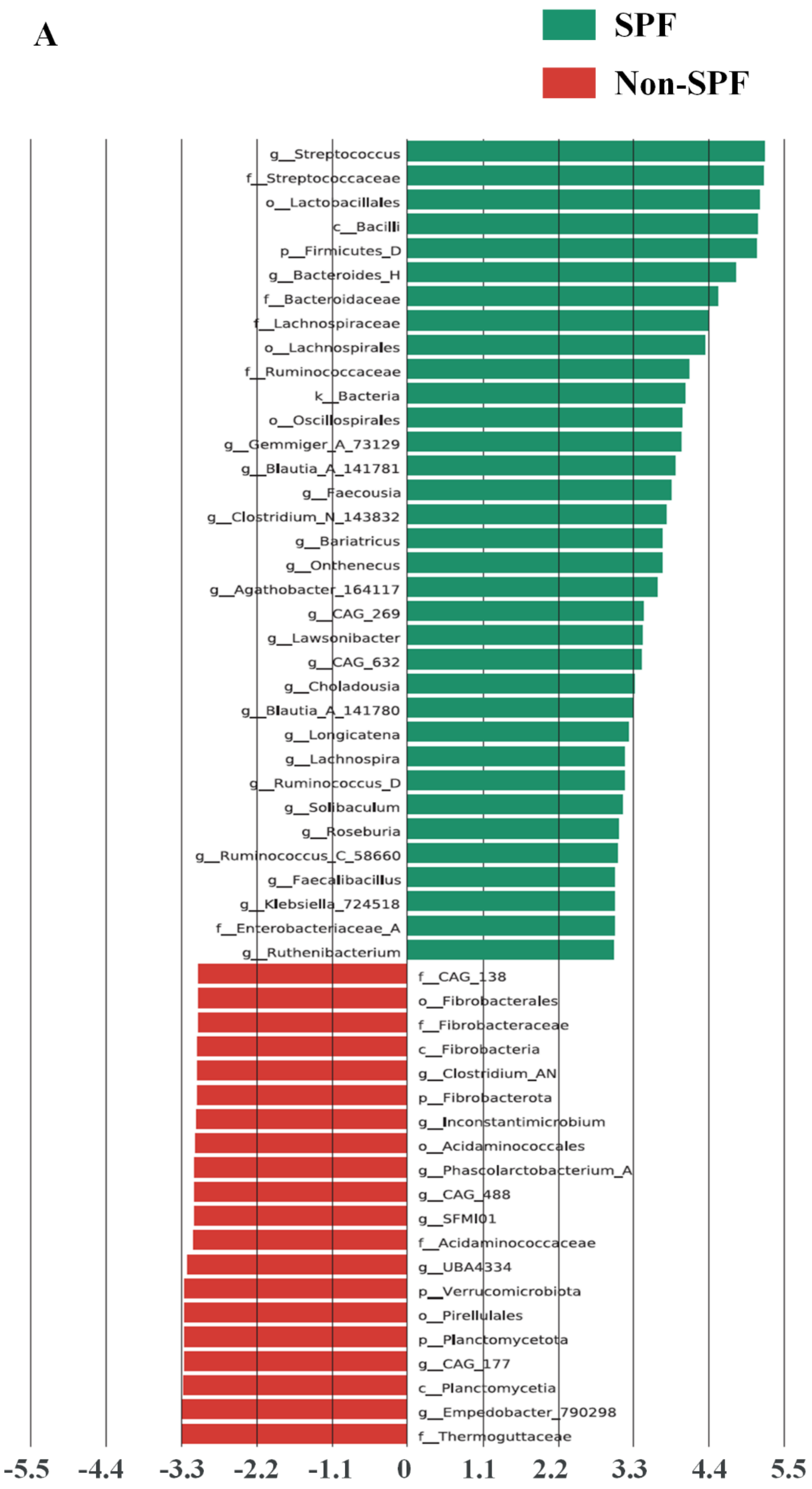

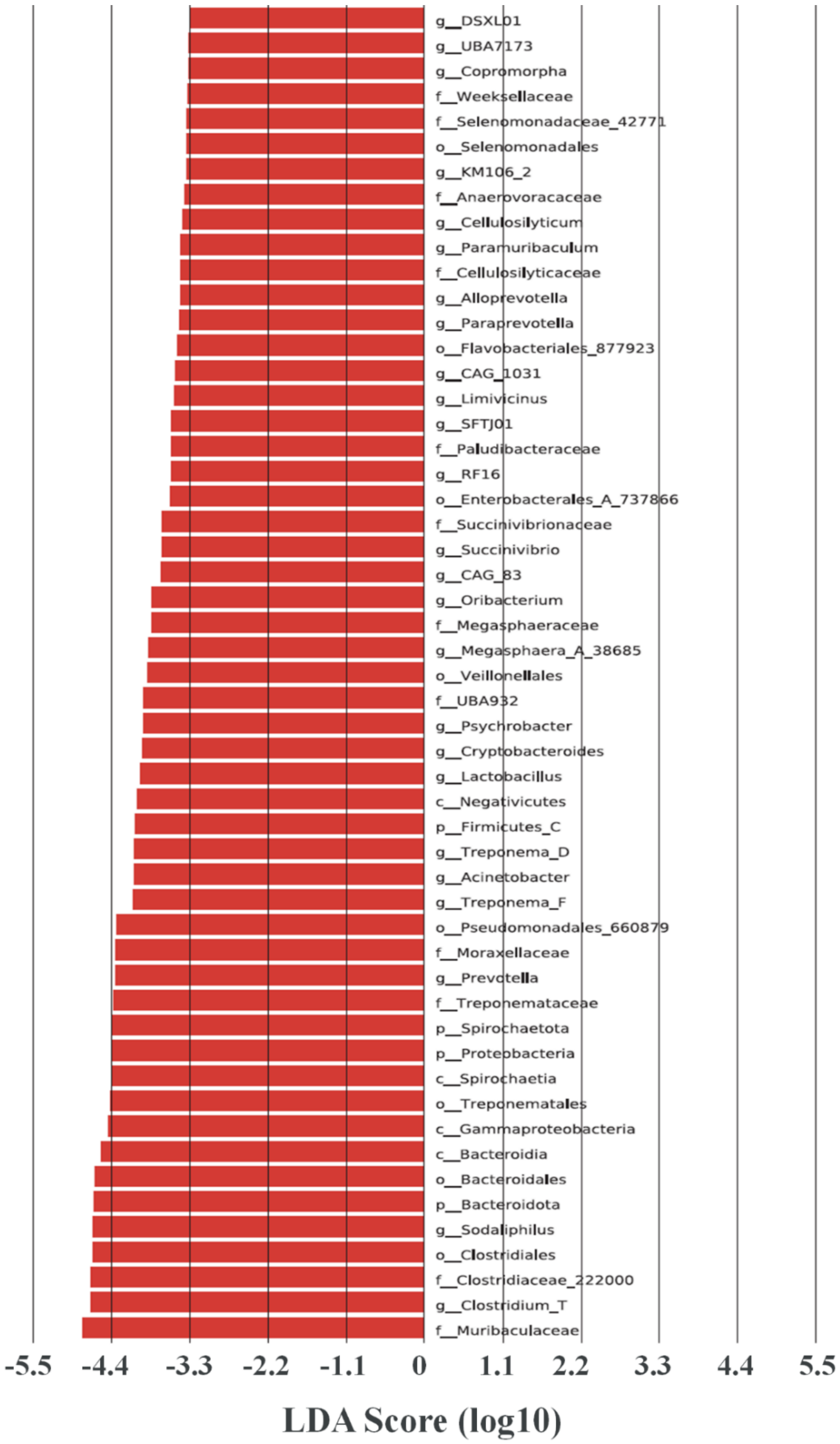
g__DSXL01
g__UBA7173
g__Copromorpha
f__Weeksellaceae
f__Selenomonadaceae_42771
o__Selenomonadales
g__KM106_2
f__Anaerovoracaceae
g__Cellulosilyticum
g__Paramuribaculum
f__Cellulosilyticaceae
g__Alloprevotella
g__Paraprevotella
o__Flavobacteriales_877923
g__CAG_1031
g__Limivicinus
g__SFTJ01
f__Paludibacteraceae
g__RF16
o__Enterobacterales_A_737866
f__Succinivibrionaceae
g__Succinivibrio
g__CAG_83
g__Oribacterium
f__Megasphaeraceae
g__Megasphaera_A_38685
o__Veillonellales
f__UBA932
g__Psychrobacter
g__Cryptobacteroides
g__Lactobacillus
c__Negativicutes
p__Firmicutes_C
g__Treponema_D
g__Acinetobacter
g__Treponema_F
o__Pseudomonadales_660879
f__Moraxellaceae
g__Prevotella
f__Treponemataceae
p__Spirochaetota
p__Proteobacteria
c__Spirochaetia
o__Treponematales
c__Gammaproteobacteria
c__Bacteroidia
o__Bacteroidales
p__Bacteroidota
g__Sodaliphilus
o__Clostridiales
f__Clostridiaceae_222000
g__Clostridium_T
f__Muribaculaceae
-5.5
-4.4
-3.3
-2.2
-1.1
0
1.1
2.2
3.3
4.4
5.5
LDA Score (log10)

14

B

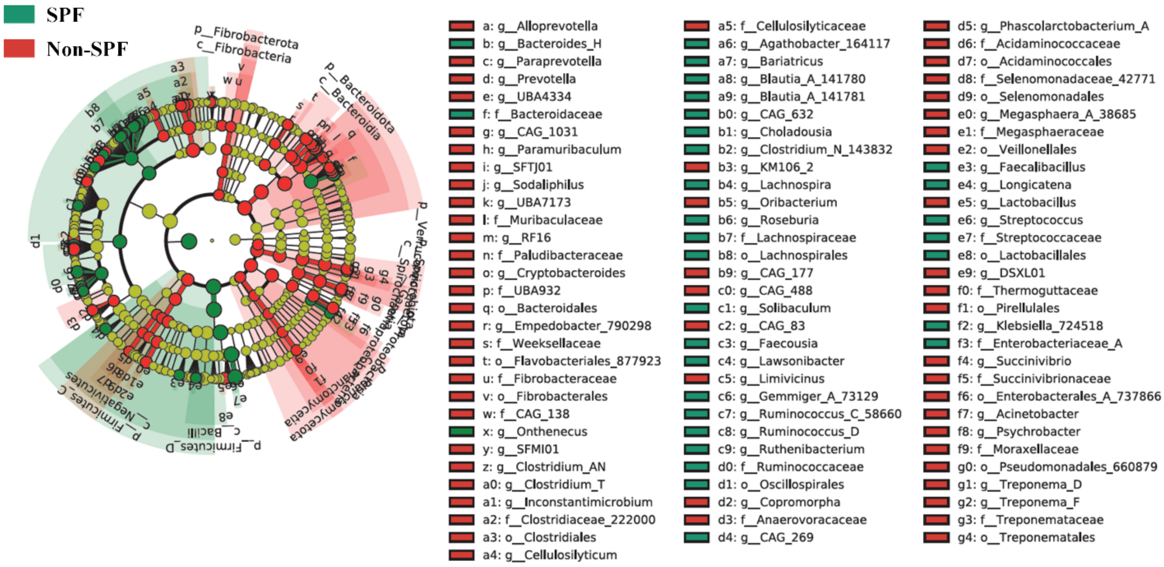

Fig. S1. Relative abundance of bacterial taxa in the gut microbiota between SPF and non-SPF pigs analyzed using linear discriminant analysis (LDA) effect size (LEfSe). (A) . Statistically significant differences are indicated by an LDA score of more than 3.0. **(B)** The cladogram shows the phylogenetic distribution of gut microbiota
